# Assessing the Potential Impact of Land Use on Carbon Storage Driven by Economic Growth: A Case Study in Yangtze River Delta Urban Agglomeration

**DOI:** 10.3390/ijerph182211924

**Published:** 2021-11-13

**Authors:** Wenyi Qiao, Weihua Guan, Xianjin Huang

**Affiliations:** 1 School of Geographic and Oceanographic Sciences, Nanjing University, Nanjing 210023, China; qiaowenyidl@163.com (W.Q.); hxjnju@163.com (X.H.); 2 School of Geographical Science, Nanjing Normal University, Nanjing 210046, China; 3 Jiangsu Center for Collaborative Innovation in Geographical Information Resource Development and Application, Nanjing 210023, China

**Keywords:** carbon storage, economic development, land use, Yangtze River Delta urban agglomeration

## Abstract

Economic development and land-use change can strongly affect terrestrial ecosystems’ carbon balance. This paper quantifies the changes in land use of Yangtze River Delta urban agglomeration (YRD) in 2020 and 2035 under three economic growth scenarios, exploring the concurrent impact on carbon storage. The results showed that the land carbon storage of YRD had decreased by 1453.80 Tg in 2000–2020, and will continue to decrease by 982.38 Tg, 1417.62 Tg, and 1636.21 Tg under the scenarios of a slow, medium, and rapid economic growth from 2020 to 2035, respectively. The large-scale occupation of cultivated land and woodland for construction land caused by economic development and population growth was an important reason. The occupation of cultivated land by construction land in Nanjing, Shanghai, and its surrounding areas had further intensified, while the reduction in carbon storage caused by the reduction in woodland had become more prominent in Hangzhou, Shaoxing, Jinhua, and the surrounding areas.

## 1. Introduction

Carbon storage in terrestrial ecosystems is a vital part of global carbon storage, profoundly influencing the carbon reduction and alleviation of global warming. Carbon storage is a process of ecosystems absorbing and accumulating atmospheric carbon [1,2]. The global terrestrial ecosystems store approximately 2030–2538 Pg of carbon, including 208–609 Pg in vegetation and 1523–1929 Pg in the top 1 m of soil [3]. As the most direct manifestation of the impact of human activities on climate change [4], land-use change is the greatest uncertainty factor in estimating carbon storage in terrestrial ecosystems. Greatly disturbed by economic growth and population agglomeration, a considerable area of the original green land has been converted into construction land [5,6,7], directly leading to a sharp decrease in land carbon storage [8,9]. Hence, there is necessary and meaningful to conduct research from the perspective of economic growth and land-use change.

There is a growing realization, at national and international levels, that we should increase carbon storage from land use to meet the challenge of carbon neutrality. In particular, land-use change can significantly alter vegetation carbon storage and soil organic carbon storage, although obvious soil organic carbon change may take longer [10]. There are differences in the carbon storage of different land-use types, and woodland usually has high soil carbon storage densities, compared with other land-use types [11,12]. Changes from woodland to other land-use types usually release carbon from the soil, especially the upper layers of soil. Hence, many scholars have proposed increasing carbon storage by adjusting the land-use structure, which appeared to be an effective plan.

Plenty of research has simulated changes in land use, but the knowledge is still limited. There is a mutual feedback relationship between land-use change and human socioeconomic development [13], and the interaction should be fully considered [14], which is often simplified or ignored. The common point of previous studies was that human activities and economic growth were regarded as external systems that affect land use, neglecting the dominant control and feedback effect of land use on human activities [15,16]. For example, Liu et al. (2017) [17] studied population, GDP, investment, and production technology as the main driving forces of land-use change and simulated the scenarios of future land use such as urban land, cultivated land, and forest land. Neoclassical economic theory believed that land also has assets and attributes that should be the input elements for economic growth [18], such as capital, labor, and technology. In other words, land is an output element as well as an input element in the urban development, rather than a one-way correlation similar to A acting on B.

A substantial proportion of the literature focused on urban land-use changes and the associated effect on carbon storage but lacked the analysis of all land-use types [19,20]. For example, He et al. (2017) [21] simulated the impact of urban ecosystems on carbon storage under different scenarios based on administrative districts and distance to the boundaries. Yang et al. (2020) [22] linked RCPs and SSPs to explore the impact of urban land-use change on carbon storage in the multiple urban expansion scenarios. However, there is a trade-off relationship between construction land and non-construction land, and a singly consideration of urban systems or non-urban systems may not reflect the current carbon storage and will thus greatly affect the precision.

The objective of this paper is to calculate the land-use change under different economic growth scenarios and analyze its impact on carbon storage, using the Yangtze River Delta urban agglomeration (YRD), China, as an example. There are two main reasons why YRD was selected as the research case. First, YRD is the sixth-largest urban agglomeration in the world and the largest urban agglomeration in China, which is undergoing a fast urbanization process with dramatic land-use change [23]. Second, the urban land-use intensity keeps increasing due to the population and industrial agglomeration, and other energy consumption-related human activities. Hence, YRD provides an ideal case to investigate the issue of changes in carbon storage caused by economic growth. Detailed research contents will include the following: (1) simulate the land-use changes of the YRD region under different economic growth scenarios from 2000 to 2030; (2) calculate the carbon storage loss caused by land-use changes; (3) provide detailed policy and strategy references from multiple angles of land-use management.

The innovation of this paper has three aspects. First, the paper developed a comprehensive study of closed loops, fully connecting social, economic, and land-use systems, and understanding their dynamic interactions and feedback. Second, the construction land and the non-construction land were included in the research framework, clarifying the structural changes of land use. Third, three economic growth scenarios were designed according to different combinations of birth rate, food demand, and land-use policy.

The structure of this paper is as follows: Section 2 describes data resources and methodology. Section 3 presents the results. Section 4 and Section 5 show the discussion and conclusions, respectively.

## 2. Methodology and Data

### 2.1. Study Area

**Figure 1:**
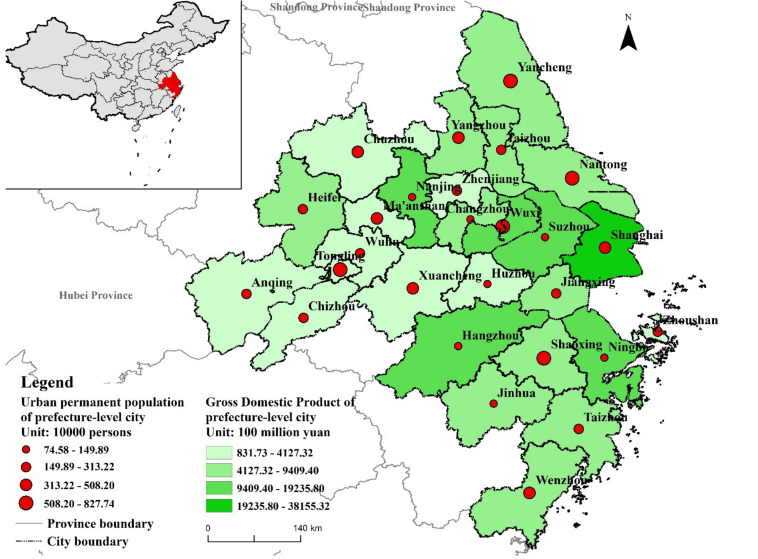
Study area.

The Yangtze River Delta urban agglomeration (YRD) is located in the developed coastal areas of eastern China, with longitude ranging from 115°46′ E to 123°25′ E and latitude ranging from 27°03’ N to 34°28’ N. It is covered Shanghai, Jiangsu, Zhejiang, and Anhui Provinces. According to the Outline of the Yangtze River Delta Regional Integrated Development Plan (2019), YRD includes 27 prefecture-level cities with an area of 225,000 km^2^ (Figure 1). Jiangsu and Zhejiang Provinces include 9 cities, respectively. Additionally, Anhui Province includes 8 cities. The economic growth and high urbanization rate in this region are accompanied by excessive population density and high demand for construction land, with frequent land-use changes.

### 2.2. Data

Data sources used in this paper include socioeconomic data, land use images, and some spatial geographic data.

Crop yield data and other socioeconomic data were obtained from statistical yearbooks of Jiangsu Province, Zhejiang Province, Anhui Province, and Shanghai, from 2001 to 2021. In order to eliminate the impact of prices, all the economic indicators involved were converted into 1990 prices.

Land use images were obtained from the Institute of Geographic Sciences and Nature Resources Research (Available online: http://www.resdc.cn/ (accessed on 22 September 2021)), with an accuracy of 30 m from 2000 to 2020. Land-use classifications used in this paper include the 6 first-level types, of cultivated land, woodland, grassland, water area, construction land, and unused land. The comprehensive valuation accuracy of the first level of land use was >93%, and that of the second level was >90% [24]. The MODIS NDVI images of a 500 m grid come from the Geospatial Data Cloud (Available online: http://www.gscloud.cn/ (accessed on 22 September 2021)).

Moreover, some spatial geographic data were collected from the database of the Chinese Academy of Sciences (Available online: http://www.resdc.cn (accessed on 22 September 2021)), such as administrative boundaries, rivers, roads, settlements, digital elevation, and soil type data, which were mainly used to identify urban development and limited regions.

### 2.3. Methods

**Figure 2:**
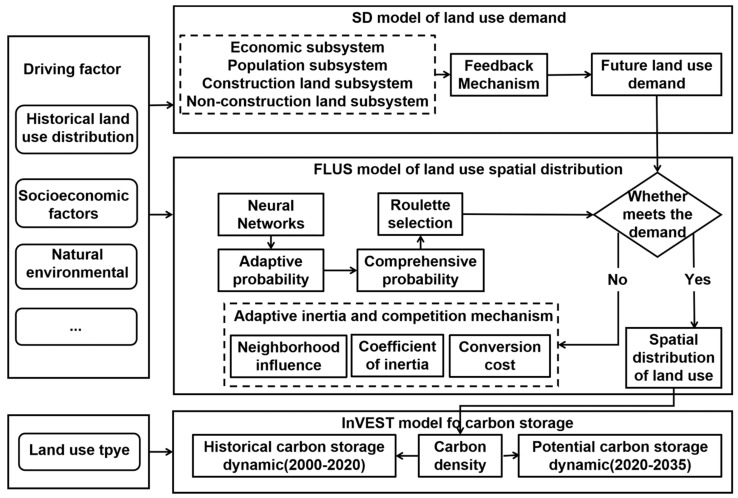
The hierarchical analysis framework.

Factors related to economic growth and urbanization will directly or indirectly affect the structure and spatial pattern of land use, leading to changes in carbon storage [25,26,27]. Figure 2 presents the research framework. The system dynamics model (SD) and future land-use simulation (FLUS) model were utilized to simulate the future land-use demand and the distribution of the spatial pattern under different coupling scenarios, respectively [17]. Additionally, the integrated valuation of ecosystem services and trade-offs (InVEST) model was used to evaluate the response of carbon storage at the land patches.

#### 2.3.1. Prediction of Future Land Use Demand

The SD model was utilized to establish the feedback relationship between urbanization, economic growth, and land use due to the obvious advantages in dealing with the nonlinear behavior of complex systems on a macro scale [28]. In the SD model, the population growth, economic development, and other relevant urbanization factors were used as driving variables to carry out multi-scenario simulations, by combining land use and industrial growth, investment, economic output per unit area of land, housing supply, and agricultural production.

**Figure 3:**
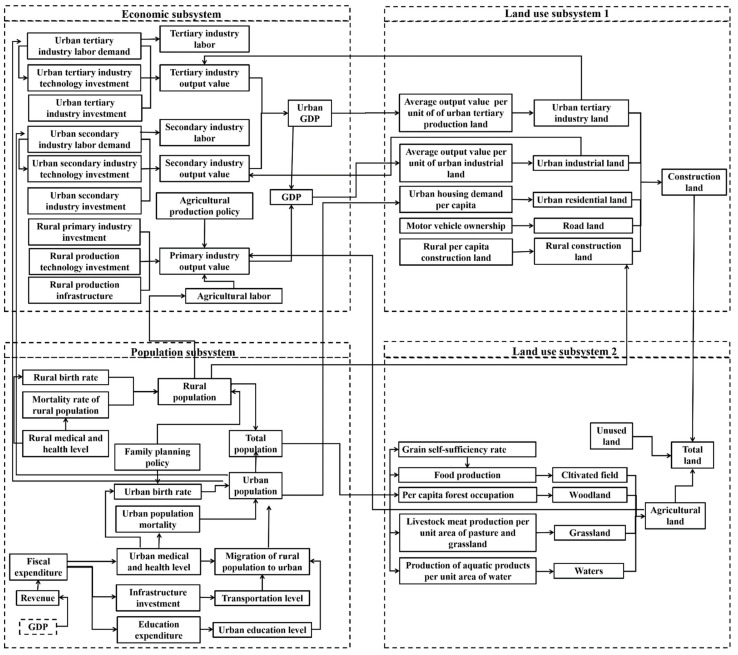
The structure of the SD model.

**Figure 4:**
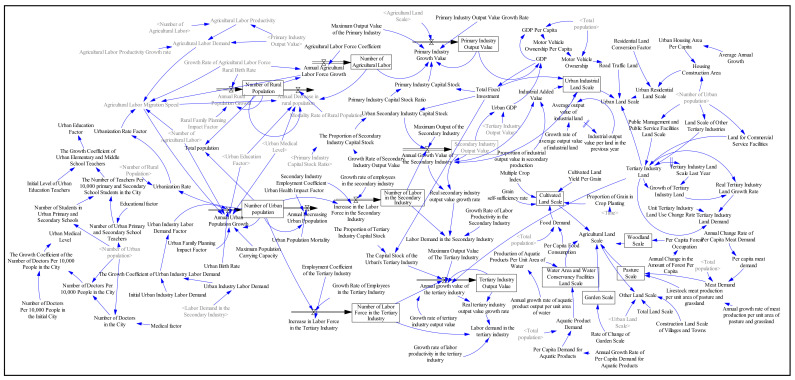
SD model of land use.

Four subsystems were used to describe the SD model, including economic subsystem, population subsystem, construction land use subsystem, and non-construction land-use subsystem, with more than 137 variables. Figure 3 shows the main variables and relationships of the four subsystems. Figure 4 provides the causal loop of the SD model.

The population subsystem mainly included changes in urban population and rural population. For example, both the continuing migration of the rural population to the city and the increasing of urban inhabitants will trigger more land demand for housing, transportation, and industrial land [29], and it also affect the structure of non-construction land through changes in dietary structure, such as the intake of fish and milk. The economic subsystem mainly included changes in the output value of the primary (secondary and tertiary) industries, which will be affected by population growth, technological development, and industrial structure adjustment. Both population subsystem and economic subsystem affected structure and amounts of land use. The increase in construction land usually led to a decrease in non-construction land, such as the loss of cultivated land, woodland, and waters.

The Vensim PLE software (Massachusetts Institute of Technology, Cambridge, MA, USA) was used to design the causal cycle diagram of the SD model. The SD model simulations begin with 2000, calibrated with 2000 and 2020 data. Table 1 shows that the errors of simulation results were less than 10%, indicating that the SD model was reliable and can be used to simulate the future land-use demand [13].

#### 2.3.2. Simulation of Spatial Land-Use Patterns

FLUS model was utilized to generate the spatial pattern of land use [17]. This paper obtained the combined conversion probability of land-use type k in grid unit p at time t by calculating the probability of occurrence, land-use conversion inertia, neighborhood effect, and land-use conversion cost. The formula is as follows:

(1) $$TP_{p,k}^{t}=P_{p,k}\times \Omega_{p,k}^{t}\times Inertia_{k}^{t}\times \left(1-sc_{c\to k}\right)$$

where $TP_{p,k}^{t}$ is the combined conversion probability. $P_{p,k}$, $\Omega_{p,k}^{t}$, $Inertia_{k}^{t}$, and $sc_{c\to k}$ represent the probability of occurrence, the neighborhood effect, the conversion inertia of land-use type k, and conversion cost from land-use type c to k, respectively.

Roulette selection was utilized to determine the land-use type k of the grid unit p after estimating the combined probability. The higher the combined conversion probability of land-use type is, the higher is the probability of being allocated to the grid unit p. This ensures the uncertainty of the actual land-use dynamic change and also reflects the randomness of the land-use spatial pattern distribution.

A three-layer ANN model was used to measure the probability of occurrence. In the input layer, each neuron corresponds to a spatial variable, including climatic factors, natural environmental factors, socioeconomic factors, and neighboring factors. Additionally, in the hidden layer, the artificial neural network was trained based on sampling data (random sampling strategy, sampling rate: 10%), combined with land-use types and other geographic information attributes. The formula is as follows:

(2) $$P_{p,k}=\sum_{j}w_{j,k}\times \frac{1}{1+e^{-net_{j}\left(p,t\right)}}$$

where $w_{j,k}$ is an adaptive weight. $net_{j}\left(p,t\right)$ is the signal received by neuron j.

The neighborhood effect reflected the interaction between the land in each grid and its surrounding grid. The formula is as follows:

(3) $$\Omega_{p,k}^{t}=\frac{\sum_{N\times N}con\left(c_{p}^{t-1}=k\right)}{N\times N-1}\times w_{k}$$

where $w_{k}$ is the weight of land-use type k. $\frac{\sum_{N\times N}con\left(c_{p}^{t-1}=k\right)}{N\times N-1}$ represents the number of grid units belonging to land-use type k in the N×N units (here, N=3) at iteration time t−1.

Land-use conversion inertia was the core of the adaptive inertia competition mechanism [30]. The formula of the conversion inertia is as follows:

(4) $$Intertia_{k}^{t}=\left\{\begin{array}{l} Intertia_{k}^{t-1},\left|D_{k}^{t-1}\right|\leq \left|D_{k}^{t-2}\right|\\ Intertia_{k}^{t-1}\times \frac{D_{k}^{t-2}}{D_{k}^{t-1}},D_{k}^{t-1}<D_{k}^{t-2}<0\\ Intertia_{k}^{t-1}\times \frac{D_{k}^{t-1}}{D_{k}^{t-2}},0<D_{k}^{t-2}<D_{k}^{t-1} \end{array}\right.$$

where $D_{k}^{t-1}$ and $D_{k}^{t-2}$ represent the difference between the macro demand and the allocation amount of land-use type k at iteration time t−1 and t−2, respectively.

#### 2.3.3. Assessment of Carbon Storage Change

This paper used the InVEST model to calculate the carbon storage of land use based on the land-use raster map, including above-ground carbon storage (AGC), below-ground carbon storage (BGC), soil organic carbon density (SOC), and dead organic matter carbon density (DOC) [31]. The formula is as follows:

(5) $$C_{p,k}=A\times (D_{AGC}^{k}+D_{BGC}^{k}+D_{SOC}^{k}+D_{DOC}^{k})$$

where $C_{p,k}$ is the carbon storage of land-use type k in unit p. A is the area of a grid cell. $D_{AGC}^{k},D_{BGC}^{k},D_{SOC}^{k}$ and $D_{DOC}^{k}$ represent the density of AGC, BGC, SOC, and DOC of the land-use type k, respectively. The values of carbon density were estimated based on the existing literature (Table 2).

### 2.4. Scenario Setting

To compare the evolution of land use in YRD under different scenarios from 2020 to 2035, 10 parameters were used: primary (secondary, tertiary) industry growth, family planning impact factor, urban housing area per capita, the change rate of industrial output value per area, grain self-sufficiency rate, the annual change rate of per capita forest occupancy and annual growth rate of livestock meat production per unit of pasture. Three development models were designed: high-speed economic growth scenario (HE), the medium economic growth scenario (ME), and the slow economic growth scenario (SE), as shown in Table 3.

#### 2.4.1. Economic Growth Rate

In order to achieve high-quality urbanization, the potential economic growth rate in the future may continue to decline [38,39], which had become the consensus of scholars and research institutions at home and abroad [40,41]. The future growth rates of the primary, secondary, and tertiary industries in YRD were determined by historical growth trends and related forecasts. Specifically, the growth rates of the primary, secondary, and tertiary industrial output values were estimated to be 5%, 7%, and 12% in high-speed economic growth scenarios, respectively. Additionally, in the moderate economic growth scenario, they were 4.5%, 6.5%, and 11.5%, respectively, while in the low-speed economic growth scenario, they were 4%, 6%, and 11%, respectively.

#### 2.4.2. Family Planning Changes

Although China had implemented the three-child policy, the trend of negative population growth was unavoidable. According to the birth data of one, two, and three children and above in the China Population and Employment Statistical Yearbook since 1998, we found that different family planning policies have different effects on the fertility rate, and the average impact coefficient was between 1.40 and 1.81. In addition, the planning document also provided an outlook on the future population’s fertility rate. For example, the “National Population Development Plan (2016–2030)” set the total fertility rate of 1.8 as the expected development target. The prediction results of the low, medium, and high total fertility rates of 2020–2050 by the United Nations “World Population Outlook 2019” were 1.4–1.52, 1.7–1.75, and 1.89–2.09, respectively [42]. The World Bank estimated that the total fertility rate between 2020 and 2050 will be approximately 1.7 to 1.8 [43]. Based on the above results, this paper assumed that the low, medium, and high total fertility rates were 1.4, 1.50, and 1.60.

#### 2.4.3. Demand for Non-Construction Land

The setting of relevant parameters for non-construction land mainly involved changes in waters, woodland, and cultivated land. In the future, the overall water area had not changed much to meet the needs of urban and rural residents for high-quality aquatic products and beautiful water resources. Therefore, this paper set the annual growth rate of aquatic product output per unit area of water in each scenario to be 3.0%, 3.2%, and 3.4%, respectively, combined with historical data.

According to the data from the sixth to ninth consecutive forest resource inventories in China, the national per capita forest land occupation has increased by approximately 0.34% per year. Therefore, the change rate of per capita forest occupancy in each scenario was set to be 0.45%, 0.30%, and 0.15%, respectively.

The amount of cultivated land must adhere to the basic principles of ensuring food security. Determining the goal of food self-sufficiency had become the basis for assessing the country’s food security status and adjusting agricultural-related policies. Generally, a food self-sufficiency rate of more than 100% is completely self-sufficient, 95% to 100% is basically self-sufficient, 90% to 95% is an acceptable level, and less than 90% indicates that food security is facing a greater risk [44]. Some scholars believed that it is more reasonable to set the goal of food self-sufficiency at around 90% in the future. Combining with the goal of stabilizing the grain self-sufficiency rate above 95% in the “Outline of National Food Security Mid-Term and Long-Term Plan (2008–2020)” [45,46], this paper set the food self-sufficiency rate as 90%, 95%, and 100%, respectively.

#### 2.4.4. Demand for Construction Land

It was expected that the per capita housing area in urban areas will continue to increase. On the one hand, there will still be a large number of people flowing from rural to urban areas in the future, generating housing demand. On the other hand, both the increasing trend of family miniaturization and improved housing can put tremendous pressure on housing land. The Evergrande Research Institute uses South Korea and Russia’s per capita housing demand as its benchmark target and assumes that China’s urban per capita housing area will increase by 1.30 to 1.50% annually from 2019 to 2030 [47].

In addition, in terms of urban industrial land, it was assumed that the output value per unit area of urban industrial land and urban tertiary industry land will continue to increase. Combined with historical data, this paper set the annual change rate of the average output value of urban industrial land in the future will increase by 0.10–0.12% annually.

## 3. Results

### 3.1. Changes in Land Use

Only the area of construction land, waters, and unused land increased during 2000–2020. Specifically, the area of construction land increased by 12,838.34 km^2^, with an average annual growth rate of 2.90%, which was primarily converted from woodland and cultivated. On the contrary, the total area of cultivated land, woodland, and grassland lost 12,781.44 km^2^, 802.90 km^2^, and 167.49 km^2^, respectively.

**Figure 5:**
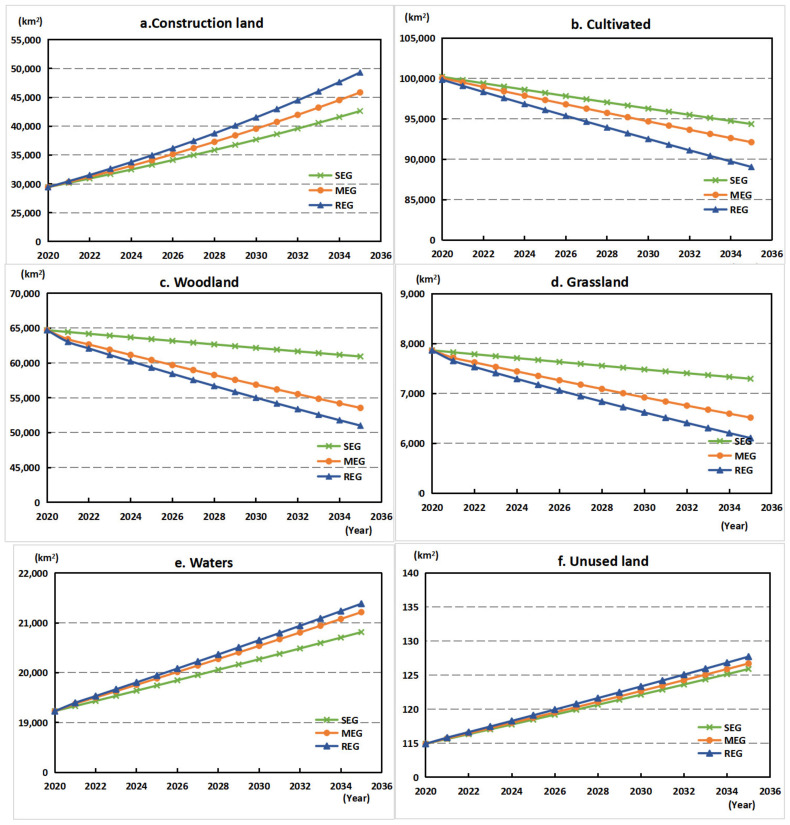
Changes in land-use quantities under different scenarios. (**a**) Construction land; (**b**) Cultivated; (**c**) Woodland; (**d**) Grassland; (**e**) Waters; (**f**) Unused land.

Figure 5 presents the quantitative changes of different land-use types under three economic growth scenarios from 2020 to 2035. During the period of 2020–2035, construction land would maintain growth, increasing by 13,193.99 km^2^, 16,421.71 km^2^, and 19,876.39 km^2^ under the scenarios of low, medium, and high economic growth. The growth rate of construction land under the high economic growth scenario was the most dramatic, which was 1.68 times that in 2020. Similar to the changing trend from 2000 to 2020, the area of cultivated land and woodland were still facing different degrees of reduction. The average annual decline rates of cultivated land under the three scenarios were 0.40%, 0.55%, and 0.76%, respectively, and the decline rates of woodland were 0.40%, 1.25%, and 1.57%, respectively. From 2020 to 2035, the area of unused land and waters will increase slightly but not significantly.

**Figure 6:**
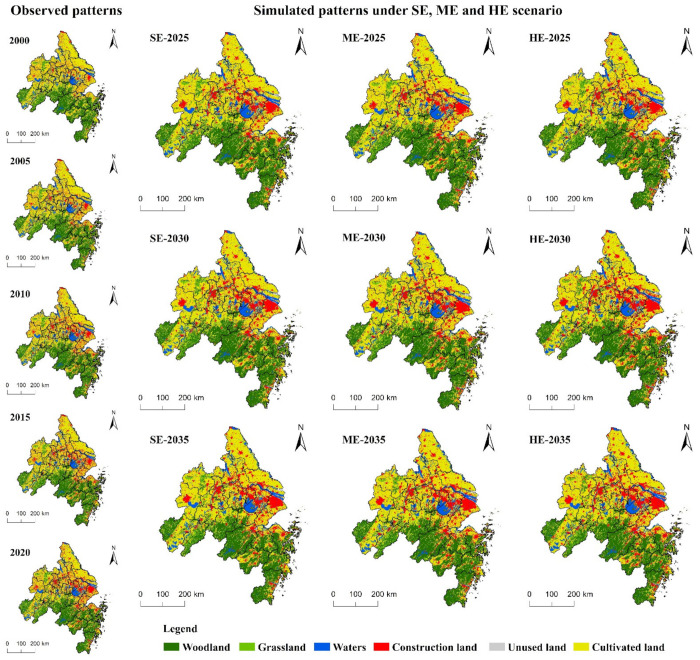
Land-use and -cover change in YRD from 2000 to 2035.

Figure 6 shows the changes in the land-use pattern of YRD from 2000 to 2035. The spatial distribution of land use in YRD had the following characteristics: (1) most newly developed construction land was concentrated in Hefei, Shanghai, northern Jiangsu, and northern Zhejiang Provinces. The production factors in these places were very concentrated, accompanied by obvious market forces. The Suzhou-Changxi metropolitan area, Hangzhou metropolitan area, and Ningbo metropolitan area were still the centers of rapid development of the YRD region, requiring a lot of construction land; (2) with the deepening of the integration process of the YRD region and its industrial transformation, cities such as Nantong and Taizhou in Jiangsu Province, as well as Wuhu and Xuancheng in Anhui Province, gradually became key development cities in the region, undertaking the transfer of industries and population. Therefore, construction land in this area showed a clear growth trend after 2010; (3) the southwest was dominated by the conversion of woodland and grassland, resulting in the concentration of land-use types with carbon sink functions in the south.

### 3.2. Potential Impact on Carbon Storage

**Figure 7:**
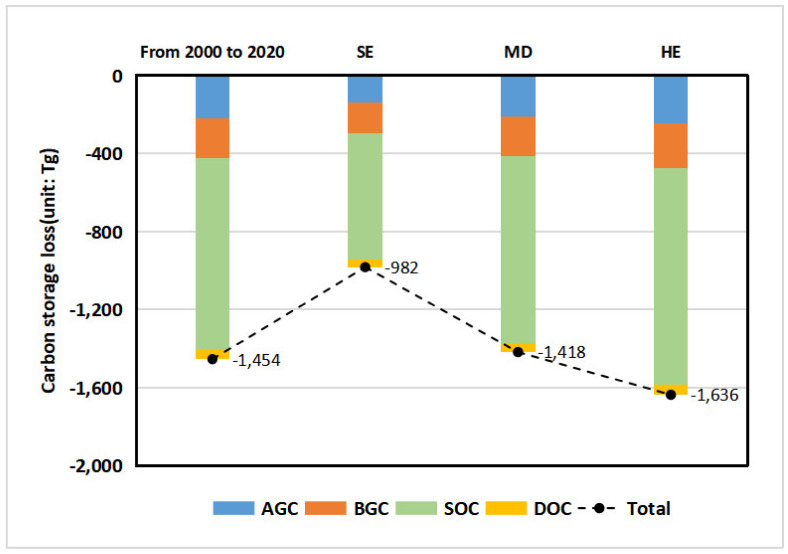
Carbon storage loss in YRD under different scenarios.

Figure 7 shows the results of changes in carbon storage from 2000 to 2035. During the process of urbanization in YRD, many woodlands, grasslands, and cultivated lands were forced to convert to construction land with negligible carbon storage capacity, resulting in a loss of carbon storage. The total scale of carbon storage in YRD was 35,809.44 Tg in 2000, and it was reduced by 1453.80 Tg in 2020, with an average annual loss rate of 0.21%. Although the average annual loss rate of carbon storage in 2020–2035 was slower than that in 2000–2020, the carbon storage in YRD dropped significantly. The loss of carbon storage showed obvious spatial heterogeneity under the next three scenarios, indicating that the speed of economic growth and the number of population growth will have a significant impact on carbon storage through land-use changes.

The reduction in SOC and AGC were the main type of carbon storage loss, accounting for 67.74% of the total loss from 2000 to 2020. Under the three scenarios from 2020 to 2035, carbon stocks continued to decline, and the average annual loss rate was lower than that in 2000–2020. The loss ratio varies from scenario to scenario, with 0.07%, −0.20%, and −0.03% under SE, ME, and HE, respectively. The reduction in SOC and AGC was still the main type of carbon storage loss.

**Figure 8:**
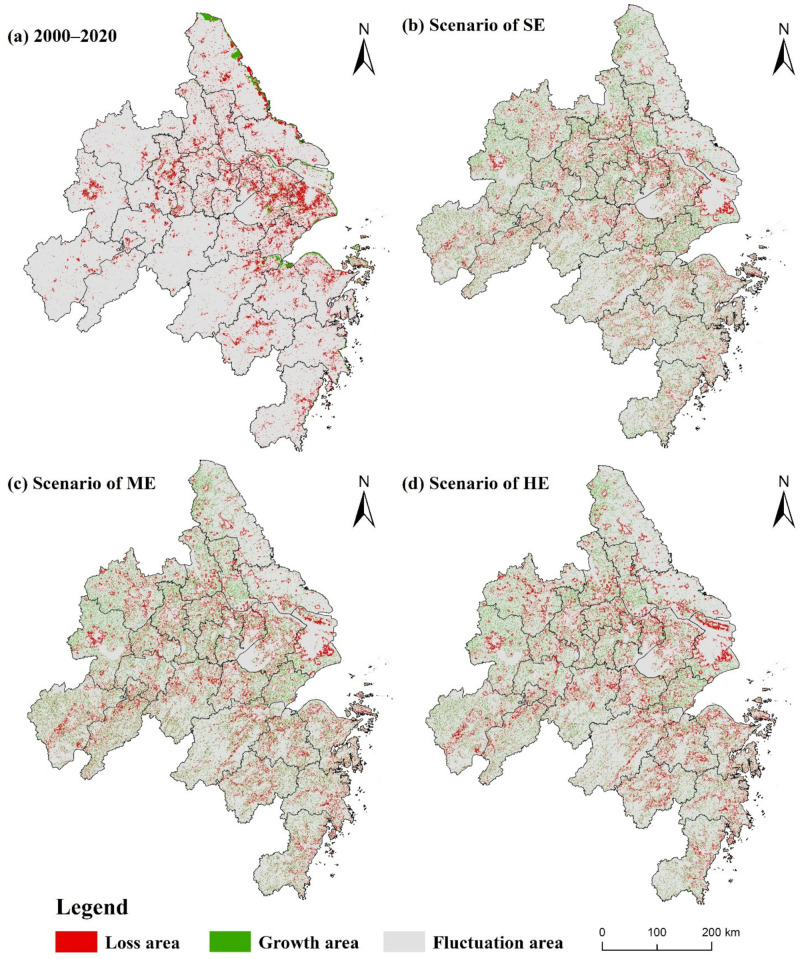
Spatial distribution of carbon storage loss in YRD: (**a**) from 2000 to 2020; (**b**–**d**) from 2020 to 2035 under the scenarios of SE, ME, and HE.

Figure 8 indicates the spatial distribution of carbon storage loss in YRD from 2000 to 2035. Changes in carbon storage also showed obvious spatial heterogeneity. From 2000 to 2020, areas with high carbon loss showed a clear Z-shaped distribution, including cities such as Nanjing, Shanghai, Suzhou, Wuxi, Hangzhou, and Ningbo. Additionally, Shanghai and its surrounding areas were experiencing more severe carbon storage loss than other regions. The second was Jiangsu Province. In addition, Hefei, Hangzhou, and its surrounding also suffered more carbon storage losses. Although carbon storage had generally declined, high-carbon storage patches were mainly distributed in the hilly and mountainous areas of the west and southeast in Zhejiang Province.

The changes in carbon storage under different economic growth scenarios were further compared. From 2020 to 2035, the carbon storage loss and gains of the YRD region would be evenly distributed. The carbon loss of Shanghai and its surrounding cities might be reduced, while the carbon storage of Anhui Province would increase significantly. Carbon storage in most regions under the scenario of slow economic growth will remain in a state of controllable fluctuations, while the scenario of high economic growth may increase or decrease substantially.

From the perspective of changes in specific cities, carbon storage still showed a significant decline in cities with high economic levels, such as Shanghai and its surrounding [48]. By contrast, the small and medium-sized cities’ carbon losses may be alleviated considering the relatively weak economic foundation and low population inflow, such as Anqing city and Chizhou city. Shanghai, in particular, would experience the most carbon storage loss. Under scenarios of a slow, medium, and high economic growth, Shanghai would experience 53.81 Tg, 74 Tg, and 95 Tg carbon storage loss, representing 5.48%, 5.24%, and 5.84% of total carbon storage loss, respectively. The second was southern Jiangsu cities, such as Suzhou and Wuxi. Under scenarios of a slow, medium, and high economic growth, Suzhou would experience 13.18 Tg, 21.86 Tg, and 29.98 Tg, carbon storage loss, representing 1.34%, 1.54%, and 1.83% of total carbon storage loss, respectively. Additionally, the carbon loss of Wuxi would be 12.25 Tg, 17.58 Tg, 22.94 Tg, accounting for 1.25%, 1.24%, and 1.40%, respectively.

This result emphasizes that policymakers should determine the main factors of carbon storage loss at the city level or even the county level, and design land-use policy and urban development plans based on local conditions. For example, protecting woodland land should be the top priority in northeastern Zhejiang, and megacities such as Shanghai should encourage the excavation of stock construction land.

### 3.3. Divergent Causes of Carbon Loss

From 2000 to 2020, the proportion of cultivated land, woodland, grassland, and unused land converted to construction land accounted for 88.10%, 6.16%, 5.70%, and 0.03% of the total newly development construction land area, and the corresponding proportion of carbon storage loss accounted for 84.89%, 6.82%, 8.28%, and 0.01%, respectively. From 2020 to 2035, the conversion of cultivated land to construction land was still the main reason for the loss of carbon storage. At the same time, the reduction in carbon storage caused by the occupation of woodland may also significantly affect some cities such as Shanghai and Jiangsu. Hence, it was very important to protect high-value carbon sink areas, and woodland protection areas should prioritize the maintenance of carbon storage as the primary task, and the mode of urban growth by occupying arable land should be encouraged as little as possible in megacities.

**Figure 9:**
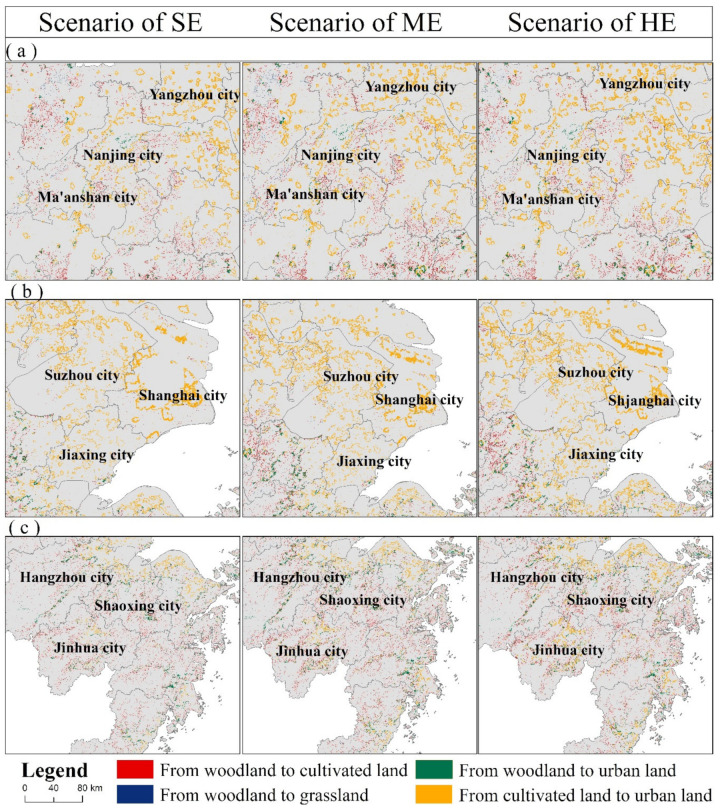
Spatial patterns of different causes of carbon storage loss from 2020 to 2035: (**a**) Nanjing, Maanshan, and Yangzhou; (**b**) Shanghai, Jiaxing, and Suzhou; (**c**) Hangzhou, Shaoxing, and Jinhua.

**Figure 10:**
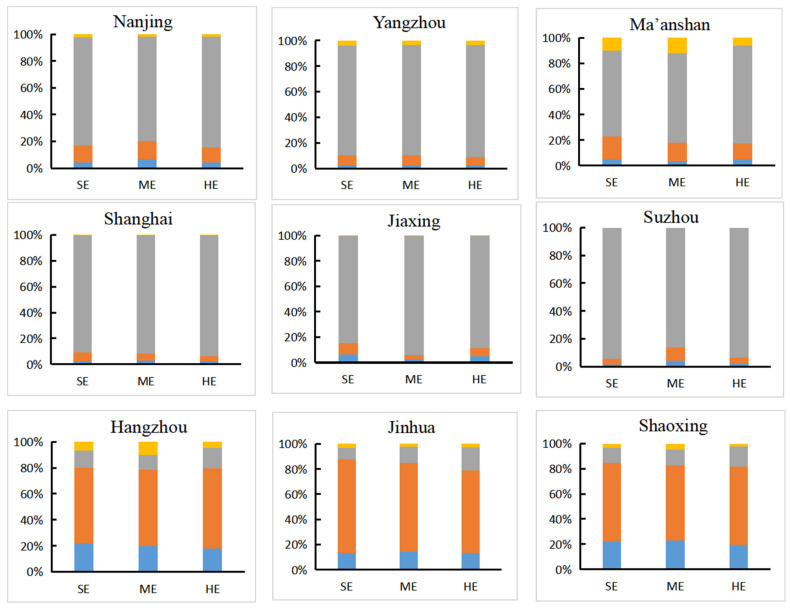
Changes in major causes of carbon loss.

Figure 9 represents the reasons for the loss of carbon storage at the urban scale of the YRD region from 2020 to 2035, which can be summarized into three types (Figure 10): (a) taking Nanjing, Maanshan, and Yangzhou as examples, the main loss of carbon storage was jointly determined by the conversion of woodland and cultivated land to construction land, and the conversion of woodland to cultivated land and grassland. Specifically, under the guidance of a series of ecological protection policies such as returning farmland to forests, the impact of deforestation for large-scale agricultural development gradually weakened, and urban expansion caused by the conversion of cultivated land gradually became the main threat of carbon storage loss; (b) for cities such as Shanghai, Jiaxing, and Suzhou, the occupation of cultivated land for construction land had always been the main reason for the reduction in carbon storage. In addition, the conversion of woodland to construction land and cultivated land was also part of the reason for the loss of carbon storage; (c) in cities with high woodland coverage such as Hangzhou, Shaoxing, and Jinhua, the conversion of woodland to cultivated land and construction land, the conversion of cultivated land to construction land, and the conversion of forest land to grassland were the basic driving forces for carbon storage. However, under the circumstances of economic growth and population increase, the carbon loss mainly caused by the conversion of woodland to farming will intensify in the future, during 2020–2035.

## 4. Discussion

### 4.1. Land-Use Change and Carbon Storage State

Urban development in coastal areas deserves more attention. The closer to the coast, the faster the urban construction land growth [49,50]. This is largely influenced by the coastal area development policy proposed by the Chinese government. Although the control of land transfer type is important, it is an inevitable trend that economic growth requires a large amount of construction land. Therefore, it is necessary to adopt other methods to increase the carbon storage of this important area, such as land management, and the combined use of inorganic fertilizer on cultivated land.

In addition, due to the decrease in the rural population, the construction land of villages and towns will enter a phase of reduction. A large area of rural residential land will likely be converted to cultivated land or other land types, which represents a huge potential for increasing carbon storage [35]. Therefore, strengthening rural land consolidation may be another effective way to increase carbon storage in the future.

### 4.2. The Comprehensive Influence on Carbon Storage

Although the carbon storage of land use in Yangtze River Delta urban agglomeration has continued to decline significantly, the annual loss rate has slowed down, compared with 2000–2020. Moreover, there is an obvious spatial heterogeneity, indicating that different economic development models can significantly change carbon storage.

Under the same socioeconomic modes, the difference in carbon loss mainly results from the increase in population. Specifically, the total population of the YRD region will reach 159.1 million by 2035, and the urbanization level will reach 81.43% under the scenario of rapid economic growth. Under the scenario of moderate economic growth, the total population will reach 19.07 million, and the urbanization level will be 81.52%. In the case of low economic growth, the total population and urbanization level are 19.07 million and 81.51%, respectively. Under the scenario of slow economic growth, the birth rate is also low, which decreases the demand for construction land. Therefore, moderate GDP and population growth will help slow down carbon storage losses [22].

Further, the growth of construction land mainly results from urban residential land, followed by industrial land and tertiary industry land. The miniaturization of family scales and the improved housing conditions of some urban residents will generate considerable housing demand [51]. Part of the demand for land development from industrial growth is offset by the increase in land output.

### 4.3. Strengths and Limitations

This paper fully considered the interdependent relationship among human activities, economic growth, and land use. The SD model of land use established can be extended to different regions. In addition, the SD model has multiple exports, such as changes in population, changes in industrial output, and changes in labor demand, which could provide the potential role of different factors in land use and carbon storage changes.

There are three limitations in the quantification of carbon density: (1) due to data limitations, the carbon density coefficients in this paper were obtained from the literature; (2) only the difference in carbon storage intensity of different land-use types was considered; (3) the change in carbon density over time was not considered, although obvious soil organic carbon change may take longer [10]. In fact, carbon density changes in time and space [52]. However, this paper discussed the changing trend of carbon storage land use driven by economic growth, rather than the precise prediction of each grid. Hence, the importance of policy recommendations in the study area will not be disturbed by the results currently presented.

The framework considered both the socioeconomic system and land-use system, in terms of both quantity and spatial distribution. However, the carbon emissions of human activities and land use were not discussed in this paper. Although these limitations do not influence the feasibility of the framework and reliability of the results, there still are many details worth to be further to discussed in land-use policy.

In addition, we acknowledge that other factors also had an impact on carbon storage, such as climate change, energy consumption, and the existence of other scenarios, such as (1) scenarios that the random combination of economic growth rate and family planning impact factors and (2) no consideration of the changes in dietary structure.

## 5. Conclusions

To the best of our knowledge, this paper is the first to assess the potential impact of land use on carbon storage driven by economic growth by examining the interdependencies between land use, economy, population, and other urbanization factors. The major conclusions are as follows: (1) from the simulations, compared with 2000–2020, the loss of future carbon storage will be effectively controlled, but it is still in a state of depletion. The land carbon storage will continue to decrease by 982.38 Tg, 1417.62 Tg, and 1636.21 Tg under the scenarios of a slow, medium, and rapid economic growth from 2020 to 2035, respectively; (2) the causes of carbon loss in different regions are heterogeneous. For example, the threat of the occupation of cultivated land by construction land in Nanjing, Shanghai, and its surrounding areas has further intensified. The carbon loss caused by the conversion of woodland in Hangzhou, Shaoxing, Jinhua, and its surrounding areas will intensify; (3) the large-scale occupation of cultivated land and woodland for construction land caused by economic development and population growth was an important reason for the loss of carbon storage.

Based on the results, three policy suggestions are proposed. First, to support a considerable population and promote economic development, built-up land expansion control faces great pressure. Therefore, other measures should be considered, for example, green-land construction among built-up land, which may also provide many other ecological functions to improve our living environment [53]. Second, controlling the population and economic growth rate should be another priority. Generally, population growth requires urban expansion to provide more settlements [10], which will result in the reduction in forest land and arable land with strong carbon sequestration capabilities. Third, it is fundamental to strengthen rural land consolidation. The Yangtze River Delta urban agglomeration is facing a considerable challenge of the loss of rural population in the future, representing that consolidation of rural residential land has great potential to increase carbon storage.

Future research may require in-depth exploration from the following aspects. First, future research needs to further explore different scenarios. Second, future research needs to improve the estimation accuracy of carbon density. In the InVEST model, carbon storage is calculated based on the four-carbon densities of different land-use types, and the spatial resolution and carbon cycle process profoundly affect the accuracy of the calculation. Third, future research needs to consider the impact of energy consumption and climate change.

## Figures and Tables

**Table 1:** Model historical data simulation error.

Main Variable	Average Error Rate
Urban population scale	1.27%
Rural population scale	1.36%
Urban land Scale	5.04%
Urban industrial land scale	0.00%
Urban residential land scale	3.55%
Land for other tertiary industries	2.48%
Cultivated land scale	−0.18%
Woodland scale	0.09%
Grassland scale	2.22%
Waters	0.16%

**Table 2:** Carbon density of each land-use type (unit: Mg/hm^2^).

Types	AGC	BGC	SOC	DOC	Source
Cultivated land	17.55	11.59	80.70	2.24	Fang et al. (2001) [32]; Jian (2001) et al. [33]; Wang et al. 2001 [34]; Chuai et al. (2013) [35]
Woodland	31.83	6.37	105.77	2.94	
Grassland	14.45	17.35	88.06	2.45	Fan et al. (2008) [36]; Chuai et al. (2013) [35]
Waters	0	0	0	0	Jian (2001) [33]; Zhang et al. (2017) [37];
Construction land	7.61	1.52	34.33	0	
Unused land	10.36	2.07	34.42	0.96	

**Table 3:** Parameters of three scenarios.

Control Variable	SE	ME	HE
Primary industry growth rate	4%	4.5%	5%
Secondary industry growth rate	6%	6.5%	7%
Tertiary industry growth rate	11%	11.5%	12%
Family planning impact factor	1.4	1.5	1.6
Annual growth of urban housing area per capita	1.3%	1.4%	1.5%
Change rate of industrial output value per area (increasing year by year)	0.10%	0.11%	0.12%
Grain self-sufficiency rate	100%	95%	90%
Annual change rate of per capita forest occupancy	0.45%	0.30%	0.15%
Annual growth rate of aquatic product output per unit area of water	3.00%	3.20%	3.40%
Annual growth rate of livestock meat production per unit of pasture	1.20%	1.40%	1.60%

## Data Availability

The data presented in this study are available on request from the author. The data are not publicly available due to privacy. Images employed for the study will be available online for readers.
